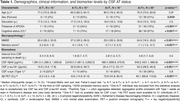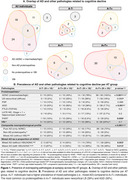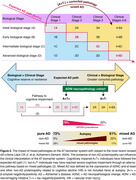# Aβ positive, tau biomarker negative cognitive impairment is associated with significant non‐Alzheimer's disease neuropathologies

**DOI:** 10.1002/alz70856_104812

**Published:** 2026-01-07

**Authors:** Konstantinos Ioannou, Richard J. Perrin, Khadidzha Abdullaieva, Marina Bluma, Konstantinos Poulakis, Antoine Leuzy, Dorota Religa, Elena Rodriguez‐Vieitez, Konstantinos Chiotis

**Affiliations:** ^1^ Department of Neurobiology, Care Sciences and Society, Karolinska Institutet, Stockholm, Sweden; ^2^ Department of Pathology and Immunology, Washington University School of Medicine, St. Louis, MO, USA; ^3^ Department of Neurology, Washington University in St. Louis School of Medicine, St. Louis, MO, USA; ^4^ Knight Alzheimer's Disease Research Center, St. Louis, MO, USA; ^5^ Wallenberg Centre for Molecular and Translational Medicine, Sahlgrenska Academy, University of Gothenburg, Gothenburg, Sweden; ^6^ Department of Psychiatry and Neurochemistry, University of Gothenburg, Gothenberg, Sweden; ^7^ Department of Psychiatry, Cognition and Aging Psychiatry, Sahlgrenska Academy, Region Västra Götaland, Sweden; ^8^ Department of Neurobiology, Care Sciences and Society, Karolinska Institutet, Stockholm, stockholm, Sweden; ^9^ Theme Inflammation and Aging, Karolinska University Hospital, Stockholm, Sweden; ^10^ Memory and Aging Center, Weill Institute for Neurosciences, University of California San Francisco, San Francisco, CA, USA; ^11^ Department of Neurology, Karolinska University Hospital, Stockholm, Sweden

## Abstract

**Background:**

This study leverages biomarker and autopsy data with the aim to evaluate the impact of mixed pathologies on interpreting cognitive impairment within the Αβ and tau (AT) biomarker system.

**Method:**

Individuals with at least one instance of antemortem CSF AD biomarkers measurement (Roche, Elecsys ®) and a full postmortem assessment were identified from the Alzheimer's Disease Neuroimaging Initiative (ADNI) database. Previously validated cut‐offs were used to define A+ (Aβ42 ≤ 981 pg/mL) and T+ (*p*‐tau181 ≥ 24.3 pg/mL). Aβ PET burden was quantified in‐house using the Centiloid pipeline. AT groups were compared with respect to cognitive performance, CSF α‐synuclein positivity (Amprion, SYNTap ®), Aβ PET burden, clinical comorbidities, and neuropathological evidence of proteinopathies associated with cognitive impairment or vascular brain injury (VBI). Mixed AD was defined as the copresence of Alzheimer's disease neuropathologic change (ADNC) and at least another non‐AD proteinopathy at autopsy.

**Result:**

We identified and grouped 77 individuals by AT status (median [IQR] interval from CSF sampling to death = 3.13 [1.45, 5.69] years). Both A+T‐ and A+T+ groups were more severely cognitively impaired than the A‐T‐ group at CSF sampling (Table 1). The A+T‐ and A+T+ groups had similar demographics, frequency of CSF α‐synuclein positivity, Aβ PET burden and frequency of clinical comorbidities. The two A+ groups showed differences in APOE ε4 frequency (79% in A+T+ vs. 22% in A+T‐, *p*‐value < 0.001) (Table 1). The A+T+ group showed a higher frequency of ADNC (100% vs. 78%, *p*‐value = 0.008) than the A+T‐ group. When ADNC was present, the A+T‐ group exhibited a higher frequency of mixed AD (79% vs. 28%, *p*‐value = 0.002) than the A+T+ group, even when taking VBI into consideration (Figure 1).

**Conclusion:**

Cognitive impairment associated with Aβ positivity and low tau (A+T‐) is linked to mixed AD. These cases deviate from the expected disease path, as proposed by the most recent criteria (Jack CR Jr, et al. Alzheimers Dement. 2024) (Figure 2), and highlight the need for specific biomarkers for non‐AD proteinopathies.